# Impact of Emergency Situations on the Level of Fear and Anxiety in Oncology Patients During Radiotherapy in a Developing Country

**DOI:** 10.7759/cureus.57129

**Published:** 2024-03-28

**Authors:** Marija Živković Radojević, Neda Milosavljević, Slobodan Jankovic, Miloš Grujić, Katarina Janković, Marko Folić

**Affiliations:** 1 Clinical Oncology, University of Kragujevac, Faculty of Medical Sciences, Kragujevac, SRB; 2 Pharmacology and Therapeutics, University of Kragujevac, Kragujevac, SRB; 3 Clinical Pharmacology, University of Kragujevac, Faculty of Medical Sciences, Kragujevac, SRB

**Keywords:** covid-19, brachytherapy, oncology patient, anxiety, fear, radiotherapy

## Abstract

Objective: To analyze the level of fear and anxiety related to radiotherapy in oncology patients treated before and during the COVID-19 pandemic, as well as to examine whether the advancement of radiotherapy centers leads to any reduction in the patient's fear in emergency situations.

Methods: Two cross-sectional studies were conducted in two time frames (2016 and 2022) based on the analysis of the intensity of anxiety and fear of radiotherapy in oncology patients with assistance. A questionnaire for assessing fear of radiotherapy in oncology patients and Zung's and Beck's self-reported anxiety scales were used. The first part of the research integrated all data of research interest obtained from patients treated with radiotherapy during 2016, and the second cross-sectional study included all patients treated in 2022 during the COVID-19 pandemic. The study was prepared according to the STROBE (Strengthening the Reporting of Observational Studies in Epidemiology) checklist.

Results: The first cross-sectional study had 154 participants who had been treated with radiotherapy, while in the second study, there were 159 patients. Patients treated in 2022 show significantly higher levels of fear and anxiety. External beam radiotherapy and brachytherapy simultaneously used in both studies increased the level of fear and anxiety.

Conclusion: The conducted research showed exceptional differences in the intensity of fear and anxiety in patients treated with radiotherapy in different health situations, as was the case during the COVID-19 pandemic, with a significant impact on the stability of the health system and the challenges to providing standard services.

## Introduction

The SARS-CoV-2 (COVID-19) pandemic has greatly affected all categories of patients. In addition to a significant share in the incidence of morbidity and mortality, it greatly influenced the functioning of the health system and contributed to an increase in fear and uncertainty among patients. Oncology patients belong to a particularly vulnerable category for several reasons: difficult access to disease diagnosis and detection, late identification of disease progression, delays in starting treatment, and frequent interruptions of oncological treatment due to infection with the SARS-CoV-2 virus, which additionally affects the outcome of treatment [1]. For these reasons, a new term was defined that refers to the onset of anxiety in patients during the COVID-19 pandemic, i.e., COVID-19-related anxiety [2].

Radiotherapy is one of the most common treatment modalities used in up to 75% of oncology patients [3]. For curative purposes, the treatment is carried out over several weeks, which increases the number of hospital visits, disrupts the patients' everyday life habits, and increases anxiety and fear. A study conducted by Živković et al., the primary goal of which was to create and validate a new questionnaire for assessing fear of radiotherapy in oncology patients (QAFRT), showed that patients' fear of radiotherapy consists of several segments [4]. A special aspect of the complex emotional experience is the fear of the impact of this treatment on the patient's relationship with family and friends, which is exacerbated by insufficient information about this therapeutic modality. A study conducted by Shaverdian et al. found that 221 (68%) of patients who underwent breast radiotherapy did not have any knowledge about radiotherapy treatment at the time of diagnosis [5]. Given that patients have difficulty understanding the ionizing radiation mechanisms of action for therapeutic purposes, the fear of potential acute or chronic side effects of radiation therapy sometimes exceeds the fear of the oncological disease itself, which can affect compliance during treatment and lead to failure to complete treatment [4,6].

A significant segment of concern is fear originating from the uncertainty of the prognosis of the disease. A meta-analysis conducted by Yang et al. showed that patients treated with radiotherapy express a higher degree of fear and anxiety about disease progression than patients treated with other therapeutic modalities (surgery and chemotherapy) [3]. This manifestation of fear strongly affects the quality of life decline and is recorded in 33% to 96% of patients [3,4].

During the second half of the last decade, we have witnessed the progressive development of radiation oncology centers in developing countries, reflected in the improvement of technical equipment, improvement in the level of staff education, and the emergence of new strategies for patient in-treatment monitoring. However, the COVID-19 pandemic has contributed to the development of fear and uncertainty among oncology patients, regardless of evident progress in this area of medicine.

The aim of the study was to perform a comparative analysis of fear and anxiety levels related to radiotherapy in oncology patients treated in 2016 and 2022, i.e., before and during the COVID-19 pandemic, as well as to examine whether the advancement of radiotherapy centers leads to any reduction in the patient's fear in emergency situations.

## Materials and methods

Study design and participants

The research was designed as a comparative analysis of two cross-sectional studies, conducted in two time frames (2016 and 2022), based on the analysis of the intensity of anxiety and fear of radiotherapy in oncology patients. The research was conducted at a tertiary center for radiation oncology in a developing country, with the approval of the Institution's Ethics Board (No.: 01/22-97). The therapeutic approach itself was carried out according to valid hospital protocols and current guidelines of good clinical practice related to the central topic of this research.

The study included all patients treated with radiotherapy at a tertiary center for radiation oncology in the period from March 1, 2022, to January 1, 2023, after signing informed consent if they met the inclusion criteria for participation in the study. The initial part of the study included relevant data from patients treated with radiotherapy in 2016, who then voluntarily filled out the QAFRT questionnaire (Appendix) and Zung's and Beck's self-reported anxiety scales, which are available for free use [7,8].

Study sampling

The first part of the research integrated all data of research interest obtained from patients treated with radiotherapy during 2016. The second cross-sectional study included all patients who had the ability to adequately understand the content of the questionnaire and answer the questions and were treated with radiotherapy during the aforementioned follow-up period in 2022.

For both the cross-sectional studies integrated into this research, the criteria for inclusion in the study were as follows: age from 18 to 85 years, pathohistologically verified malignant tumor at any stage of disease, Serbian speaker, and ability to understand the contents of the questionnaires. The criteria for exclusion from the study were: younger than 18 years and older than 85 years at the time of pathohistological verification of cancer, mental illness, inability to understand the contents of the questionnaires, incomplete medical documentation, and violation of the study protocol. The study was prepared according to the STROBE (Strengthening the Reporting of Observational Studies in Epidemiology) checklist.

Independent variables

Intensities of anxiety and fear of radiotherapy in oncology patients that were treated during 2016 and 2022 were assessed based on the validated QAFRT questionnaire and Zung's and Beck's self-assessment anxiety scales in the Serbian language.

Dependent variables

The dependent variables included medical records data (medical history and radiotherapy chart), pathohistological type of tumor, other prescribed treatment modalities (surgery, chemotherapy, immunotherapy), comorbidities according to the Charlson comorbidity score [9], the goal of radiotherapy (curative or palliative radiotherapy), method of application (external beam radiotherapy (EBRT) and/or brachytherapy), field localization, radiotherapy technique and immobilization devices used, history of infection with SARS-CoV-2 virus, and SARS-CoV-2 vaccination status.

Confounding variables

Patient sociodemographic characteristics, including gender, marital status, occupation, education, and religiousness, the presence or absence of a detailed explanation by the radiation oncologist regarding the therapeutic procedure itself, the expected duration of treatment, and potential acute or chronic toxicity.

Research procedure

Using patients' medical disease history and radiotherapy chart, data were obtained on the pathohistological characteristics of the tumor and the disease stage, the proposed and implemented therapeutic protocols, and the radiotherapy treatment regimen. All patients were treated in accordance with the current hospital protocol and good clinical practice guidelines for the given period, depending on the type and stage of the disease. After they had signed the informed consent, patients who met the inclusion criteria and who were scheduled for radiotherapy treatment during 2022 proceeded to fill out the QAFRT questionnaire and Zung's and Beck's self-assessment anxiety scales after the first radiotherapy fraction. The initial part of the analysis was conducted by reviewing the medical records of patients treated during 2016 who then voluntarily filled out the QAFRT questionnaire, Zung's and Beck's self-assessment anxiety scales, and were screened to ensure they met all inclusion criteria.

The QAFRT questionnaire for assessing the level of fear of radiotherapy in oncology patients consists of three parts. The first part contains questions related to the patient's sociodemographic characteristics. The second part contains information related to the disease. The third part of the questionnaire refers to the patient's fear of radiotherapy. Each question from the third part has five answers offered according to the Likert scale, marked from 0 to 4. To fill in the Zung's and Beck's scales, four possible answers are offered according to the Likert scale, marked from 0 to 3. Before filling out the questionnaire, all respondents received adequate verbal and written explanations on how to answer the questions from the questionnaire.

Strength of the study

The sample size was determined based on a study power of 80% and a probability of type 1 statistical errors (α) of 0.05. According to the formula for calculating the sample size when looking for the mean value of a continuous variable in the population, with a relevant, literature-based standard deviation of measurement (SD = ±0.94) and width of the confidence interval of d = ±0.3, it was determined that it would be necessary to include a minimum of 150 patients in the research in both studies [4,10].

Statistical methods used to process the data acquired

The collected data were processed using descriptive statistics methods using measures of central tendency and standard deviation for continuous variables with normal distribution and relative frequency for categorical variables. For continuous variables, the significance of differences was tested using the parametric Student's t-test and non-parametric tests (Mann-Whitney U test) in case of irregular data distribution. The χ2 (chi-squared) test was used for categorical variables. The difference in the compared data was considered statistically significant if the probability of the null hypothesis was less than 5% (p < 0.05). Pearson's test, Student's t-test, and ANOVA were used for the difference of scores between groups. Spearman's coefficient was used to examine the convergent correlation and check for temporal stability between scores on the QAFRT questionnaire and Zung's and Beck's scales completed in two time frames six years apart. SPSS version 18 statistical software for Windows (Chicago, IL, USA) was used for data calculation and processing.

## Results

The first cross-sectional study conducted in 2016 had 154 (100%) participants who had been treated with radiotherapy, while in the second study carried out in 2022 during the COVID-19 pandemic, there were 159 (100%) patients.

The sociodemographic characteristics of patients and QAFRT response score values are shown in Table 1. The gender distribution of patients was equivalent in both studies (Table 1). The patients treated in 2016 were slightly older (64.46 ± 10.04 years) compared to patients treated in 2022 (58.86 ± 13.86 years). The second study shows a significant reduction in patients living in a family (92 (57.9%) compared to 110 (71.4%), and an equal number of patients live in rural and urban areas. The Charlson comorbidity score values did not differ significantly between the studies. During COVID-19, a greater number of patients were treated with chemotherapy (125 (78.6%) compared to 82 (53.2%)), and significantly fewer with surgery than in 2016 (76 (47.8%) compared to 101 (65.6%)) (Table 1). Depending on the radiotherapy intention, there was no significant difference in the frequency of application of palliative or curative radiotherapy. In the first study, the only applied radiotherapy technique was the 2D conventional technique, while in 2022, this technique was used in 28 (17.6%) patients, and high-precision techniques (3D-conformal radiotherapy, intensity-modulated radiation therapy, and volumetric modulated arc therapy) dominated (Table 1). In the second study, 140 (88.1%) patients believed that they had received an adequate explanation of the treatment regimen, pre-therapeutic preparation, and potential acute and chronic complications, while in 2016, 20 (13%) patients claimed the same. Previous SARS-CoV-2 infection was reported by 92 (57.9%) patients, while 91 (57.2%) were vaccinated.

**Table 1:** Level of fear of radiotherapy in 2016 and 2022 in oncology patients * Pearson test; ** t-test; *** ANOVA. QAFRT = questionnaire for assessing fear of radiotherapy in oncology patients.

Variable	2016 (n = 154) (100.0%)			2022 (n = 159) (100.0%)		
	Mean (SD)	QAFRT score, mean (SD)	P-value	Mean (SD)	QAFRT score, mean (SD)	P-value
Gender						
Male	77 (50.0%)	11.38 (±11.64)	p=0.484^***^, F=0.492	76 (47.8%)	18.86 (±13.90)	p=0.000^***^, F=13.564
Female	77 (50.0%)	10.17 (±9,62)		83 (52.2%)	28.35 (±18.12)	
Age	64.46 (±10.04)	10.77 (±10.46)	p=0.528^*^, t=0.051	58.86 (±13.86)	23.81 (±16.87)	p=0.027^*^, t=-0.175
Education	9.81 (±3.68)	10.57 (±10.66)	p=0.715^*^, t=0.030	11.31 (±3.82)	23.71 (±16.82)	p=0.582^*^, t=-0.044
Marital status						
Single	8 (5.2%)	14.3 (±9.78)	p=0.525^***^, F=0.436	28 (17.6%)	22.11 (±16.99)	p=0.374^***^, F=0.284
Married	108 (70.1%)	10.28 (±10.27)		90 (56.6%)	24.86 (±17.60)	
Divorced	6 (3.9%)	10.00 (±9.80)		17 (8.8%)	21.86 (±16.90)	
Widow	32 (20.8%)	11.75 (±12.47)		27 (17.0%)	23.11 (±14.79)	
Community life	110 (71.4%)	10.25 (±10.18)	p=0.092^**^, t=0.890	92 (57.9%)	23.87 (±16.79)	p=0.915^**^, t=0.025
Residence						
Village	69 (44.8%)	10.99 (±10.33)	p=0.824^**^, t=-11.821	77 (48.4%)	24.49 (±16.10)	p=0.215^**^, t=-0.139
City	85 (55.2%)	10.60 (±10.99)		82 (51.6%)	23.17 (±17.64)	
Work conditions						
Office work	10 (8.4%)	8.33 (±10.21)	p=0.243^***^, F=1.129	35 (22.0%)	24.00 (±15.07)	p=0.178^***^, F=2.377
Moderate physical activity	54 (33.2%)	14.17 (±12.49)		45 (28.3%)	28.93 (±18.51)	
Hard physical work	10 (6.5%)	13.90 (±12.06)		30 (18.9%)	22.13 (±14.28)	
Retiree	80 (51.9 %)	10.05 (±11.19)		49 (30.8%)	20.00 (±16.78)	
Religiosity	129 (83.8%)	11.29 (±11.28)	p=0.048^**^, t=-12.193	130 (81.8%)	23.92 (±16.88)	p=0.613^**^, t=-0.014
Charlson comorbidity score	38.29 (±30.63)	10.77 (±10.66)	p=0.574^*^, P=0.046	39.15 (±35.01)	23.81 (±16.87)	p=0.042^*^, t=0.162
Chemotherapy	82 (53.2%)	10.35 (±10.40)	p=0.604^**^, t=0.042	125 (78.6%)	24.02 (±17.18)	p=0.770^**^, t=-0.023
Surgery	101 (65.6%)	11.50 (±11.13)	p=0.241^**^, t=-0.095	76 (47.8%)	23.00 (±17.84)	p=0.563^**^, t=0.046
Radiotherapy purpose						
Curative	125 (81.2%)	10.17 (±10.04)		121 (76.1%)	24.02 (±17.04)	p=0.785^***^, F=0.074
Palliative	29 (18,8%)	13.38 (±12.90)	p=0.191^***^, F=2.151	38 (23.9%)	23.16 (±16.51)	
Radiotherapy technique						
2D - conventional	154 (100.0%)	13.38 (±12.90)	p=0.000^***^, F=-11.452	28 (17.6%)	23.64 (±18.08)	p=0.966^***^, F=1.446
3D - conformal radiotherapy	0 (0.0%)	0.00 (±0.00)		46 (28.9%)	24.33 (±17.03)	
Intensity-modulated radiation therapy	0 (0.0%)	0.00 (±0.00)		28 (17.6%)	28.96 (±16.02)	
Volumetric modulated arc therapy	0 (0.0%)	0.00 (±0.00)		57 (35.8%)	20.96 (±16.33)	
Method of application						
External beam radiotherapy (EBRT)	147 (100.0%)	10.59 (±10.38)	p=0.036^***^, F=1.002	124 (78.0%)	21.41 (±16.18)	p=0.002^***^, F=6.236
Brachytherapy (BT^)^		0.00 (±0.00)		4 (2.5%)	28.00 (±12.54)	
EBRT + BT	7 (4.5%)	14.71 (±16.17)		31 (19.5%)	32.87 (±17.29)	
Immobilization device	17 (11.0%)	11.12 (±10.77)	p=0.888^**^, t=-0.011	61 (38.4%)	24.11 (±17.20)	p=0.890^**^, t=-0.050
Explanation of the radiation oncologist	20 (13.0%)	9.30 (±10.35)	p=0.510^**^, t=0.054	140 (88.1%)	24.03 (±17.19)	p=0.661^**^, t=-0.035
COVID-19 infection history	NA	NA	NA	92 (57.9%)	24.41 (±16.83)	p=0.600^**^, t=-0.042
COVID-19 vaccination	NA	NA	NA	91 (57.2%)	23.97 (±16.50)	p=0.923^**^, t=-0.015
Type of vaccine						
Pfizer-BioNTech	NA	NA	NA	24 (26.4%)	22.88 (±17.86)	p=0.248^***, ^F=0.041
Sinopharm	NA	NA		49 (53.8%)	24.04 (±16.93)	
Sputnik V	NA	NA		18 (19.8%)	23.83 (±17.19)	

Patients with religious convictions returned high response scores to QAFRT in 2016 (p = 0.048), as did the patients who received EBRT and brachytherapy (p = 0.036), which was also confirmed in the second study (p = 0.002). In addition, in 2022, women (p = 0.000), older patients (p = 0.027), and patients with higher values on the Charlson comorbidity score (p = 0.042) showed a higher degree of anxiety.

The results presented in Table 2 indicate that the patients treated in 2022 had higher stages of disease and significantly higher response scores on the QAFRT.

**Table 2:** Level of fear of radiotherapy in 2016 and 2022 in oncology patients according to tumor type and clinical stage * For tumors of the central nervous system, stages I, II, and III of the disease indicate the grade of the tumor, while stage IV indicates tumors that require palliative radiation therapy. n (%) = number (percent) of patients.

Clinical stage		Type of tumor										
		CNS tumors*, n (%)	Head and neck tumors, n (%)	Lung cancer, n (%)	Breast cancer, n (%)	Urogenital tumors, n (%)	Gynecological tumors, n (%)	Gastrointestinal tumors, n (%)	Skin and soft tissue tumors, n (%)	Lymphomas and leukemias, n (%)	Total, n (%)	QAFRT score, mean (SD)
I	2016, n (%)	2 (15.4%)	5 (22.7%)	4 (14.8%)	4 (11.4%)	6 (19.4%)	2 (28.6%)	1 (7.1%)	0 (0.0%)	0 (0.0%)	24 (15.6%)	12.33 (±9.37)
	2023, n (%)	2 (13.3%)	1 (6.7%)	0 (0,0%)	2 (13.3%)	2 (13.3%)	9 (60.0%)	0 (0.0%)	0 (0.0%)	0 (0.0%)	15 (9.4%)	29.47 (±21.75)
II	2016, n (%)	6 (46.2%)	14 (63.6%)	5 (18.5%)	16 (45.7%)	18 (58.1%)	3 (42.9%)	11 (78.6%)	5 (100.0%)	0 (0.0%)	83 (53.9%)	8.60 (±9.23)
	2023, n (%)	0 (0.0%)	5 (8.8%)	1 (1.8%)	13 (22.8%)	14 (24.6%)	13 (22.8%)	9 (15.8%)	0 (0.0%)	2 (3.5%)	57 (35.8%)	21.14 (±17.45)
III	2016, n (%)	3 (21.3%)	2 (9.1%)	10 (37.0%)	11 (31.4%)	6 (19.4%)	2 (28.6%)	2 (14.3%)	0 (0.0%)	0 (0.0%)	31 (20.1%)	12.74 (±11,37)
	2023, n (%)	1 (2.0%)	6 (11.8%)	6 (11.8%)	2 (3.9%)	8 (15.7%)	15 (29.4%)	12 (23.5%)	0 (0.0%)	1 (2.0%)	51 (32.0%)	23.08 (±15.68)
IV	2016, n (%)	2 (15.4%)	1 (4.5%)	8 (29.6%)	4 (11.4%)	1 (3.2%)	0 (0.0%)	0 (0.0%)	0 (0.0%)	0 (0.0%)	16 (10.4%)	15.88 (±15.35)
	2023, n (%)	5 (14.3%)	1 (2.9%)	10 (28.6%)	4 (11.4%)	9 (25.7%)	3 (8.6%)	0 (0.0%)	2 (5.7%)	1 (2.9%)	35 (22.0%)	21.49 (±15.39)
Total	2016, n (%)	13 (8.4%)	22 (13.8%)	27 (17.5%)	35 (22.7%)	31 (20.1%)	7 (4.5%)	14 (9.1%)	4 (2.9%)	0 (0.0%)	154 (100.0%)	p=0.046 (clinical stage), F=3.904
	2016 QAFRT score, mean (SD)	9.77 (±5.12)	9.91 (±5.12)	10.78 (±12.18)	11.63 (±10.25)	9.26 (±11.02)	22.43 (±17.08)	9.71 (±12.36)	7.20 (±3.90)	0.00 (±0.00)		p=0.010 (tumor types), F=2.399
	2023, n (%)	7 (4.4%)	14 (8.8%)	17 (10.7%)	21 (13.2%)	33 (20.8%)	40 (25.2%)	21 (13.2%)	2 (1.3%)	4 (2.5%)	159 (100.0%)	p=0.052 (clinical stage), F=0.816
	2022 QAFRT score, mean (SD)	21.86 (±20.36)	23.14 (±14.62)	14.59 (±13.60)	28.95 (±20.52)	17.27 (±12.93)	30.35 (±17.48)	23.57 (±13.96)	32.50 (±33.23)	27.25 (±15.17)		p=0.016 (tumor types), F=1.793

**Figure 1:**
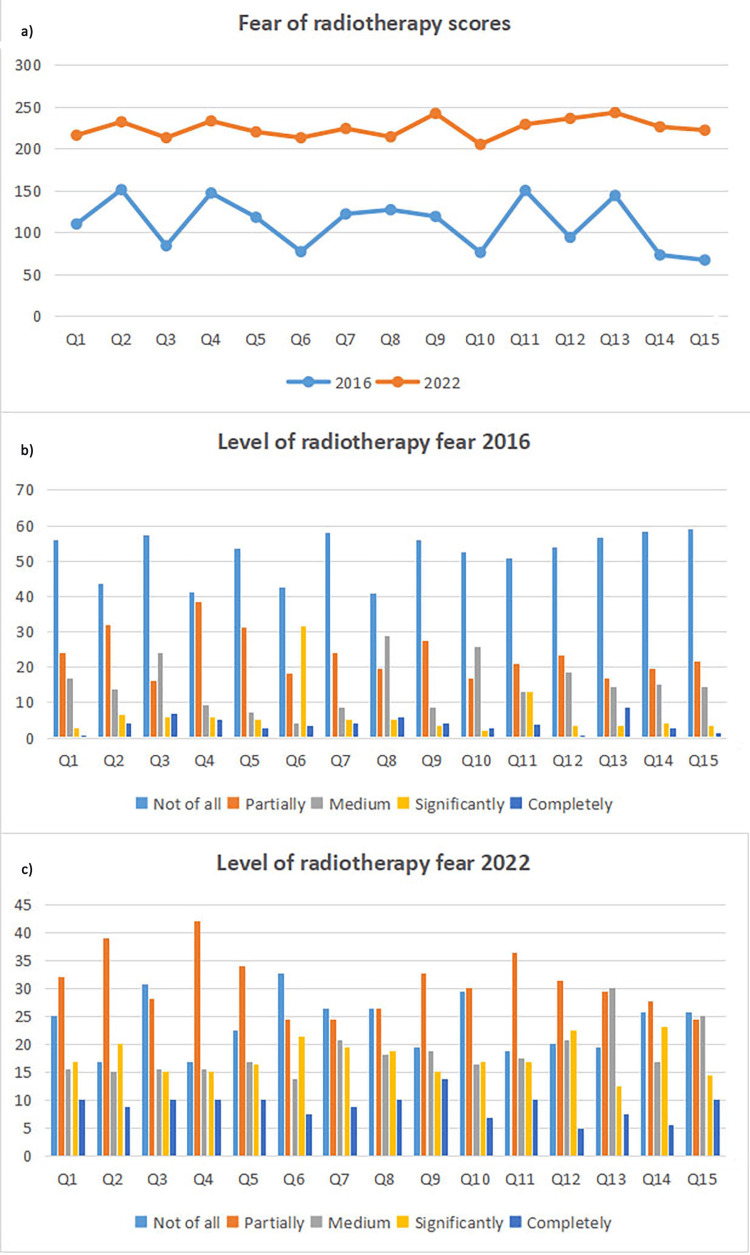
Level of radiotherapy fear according to QAFRT in 2016 and 2022 (a) The X-axis represents the total scores of the answers according to the questions (Q) order from the QAFRT filled out by the patients in 2016 and 2022, and the Y-axis represents the maximum score value for each question from the QAFRT. (b) The X-axis represents the distribution of certain answers according to the questions (Q) order from the QAFRT that patients filled out in 2016, and the Y-axis represents the number of patients. (c) The X-axis represents the distribution of certain answers according to the questions (Q) order from the QAFRT that patients filled out in 2022, and the Y-axis represents the number of patients. QAFRT = questionnaire for assessing fear of radiotherapy in oncology patients.

**Figure 2:**
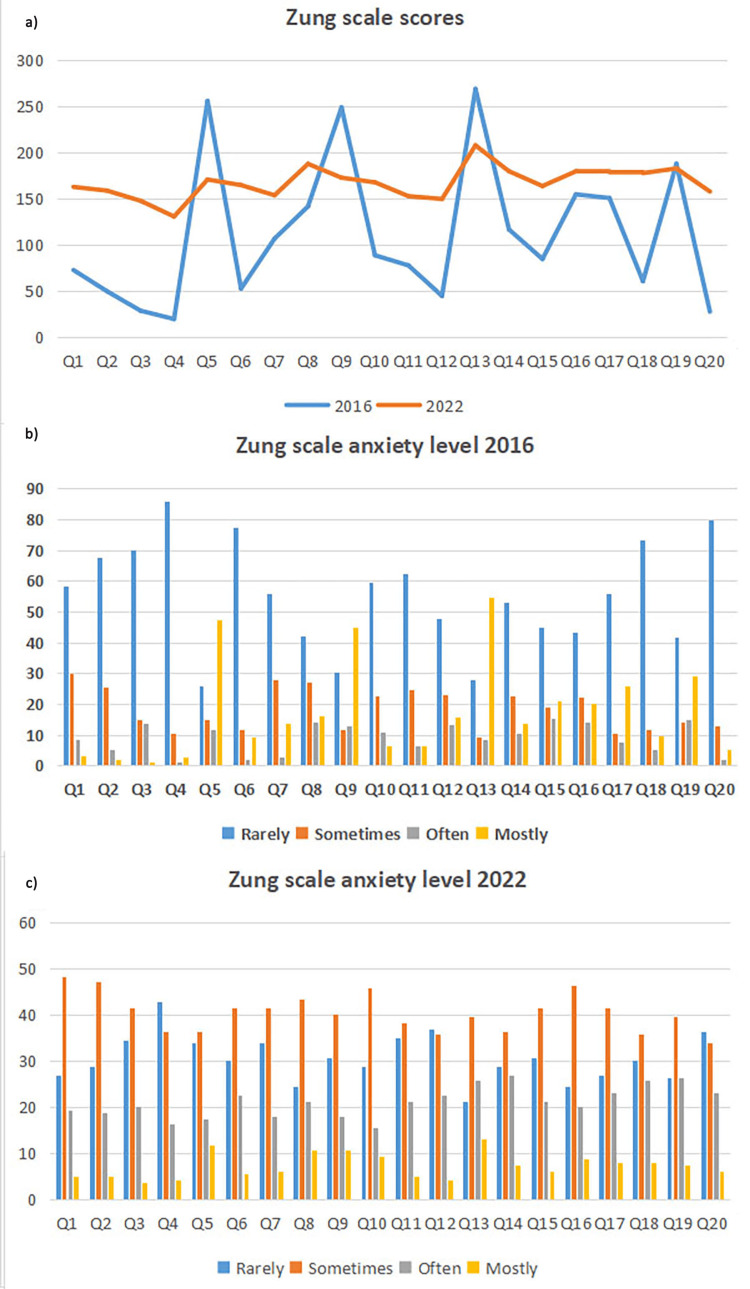
Level of anxiety according to the Zung scale in 2016 and 2022 (a) The X-axis represents the total scores of the answers according to the questions (Q) order from the Zung scale filled out by the patients in 2016 and 2022, and the Y-axis represents the maximum score value for each question from the Zung scale. (b) The X-axis represents the distribution of certain answers according to the questions (Q) order from the Zung scale that patients filled out in 2016, and the Y-axis represents the number of patients. (c) The X-axis represents the distribution of certain answers according to the questions (Q) order from the Zung scale that patients filled out in 2022, and the Y-axis represents the number of patients.

**Figure 3:**
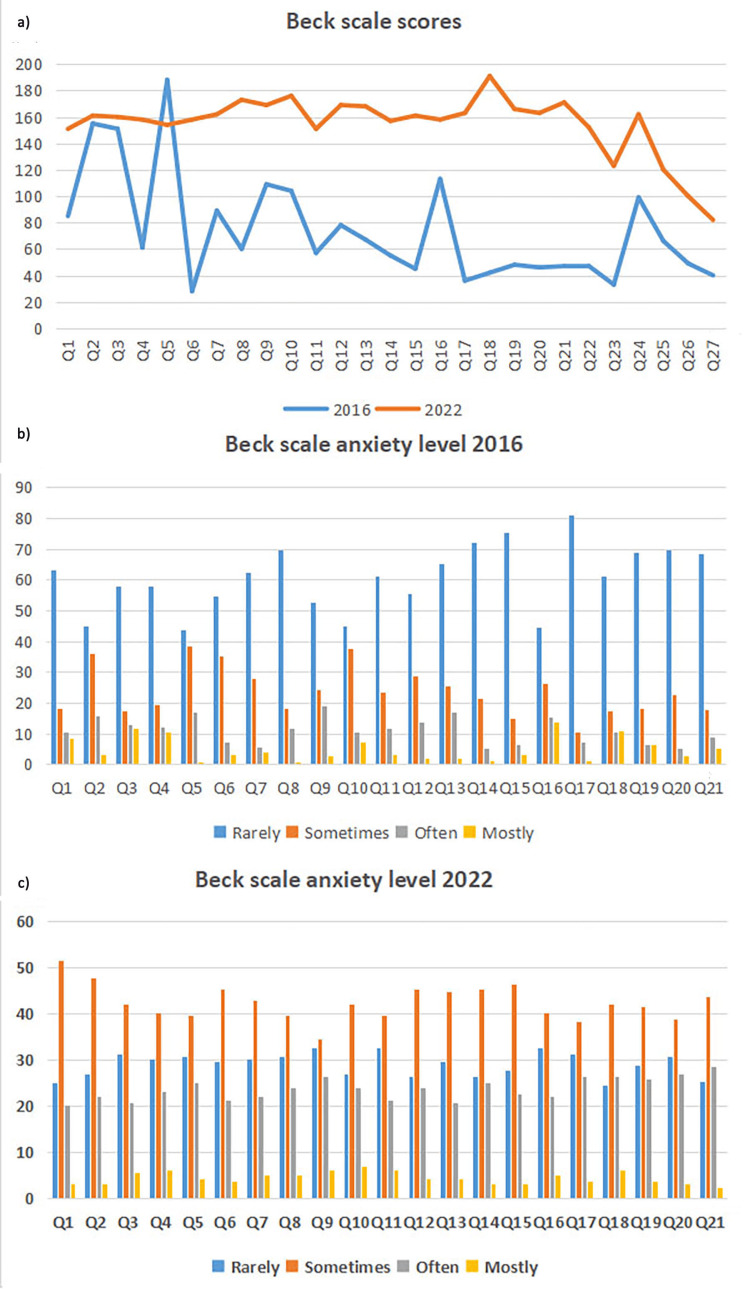
Level of anxiety according to the Beck scale in 2016 and 2022 (a) The X-axis represents the total scores of the answers according to the questions (Q) order from the Beck scale filled out by the patients in 2016 and 2022, and the Y-axis represents the maximum score value for each question from the Beck scale. (b) The X-axis represents the distribution of certain answers according to the questions (Q) order from the Beck scale that patients filled out in 2016, and the Y-axis represents the number of patients. (c) The X-axis represents the distribution of certain answers according to the questions (Q) order from the Beck scale that patients filled out in 2022, and the Y-axis represents the number of patients.

The total scores on the questions, as well as the response distribution according to the Likert scale from the QAFRT questionnaire and the Zung and Beck scales in the two time frames are presented in Figures 1-3. Additional analysis showed an exceptional convergent correlation between the response scores on the questions for all three scales in the years when the studies were conducted. However, no correlation was confirmed for all the scores of all three scales when looking at the results from 2016 and 2022 (Table 3).

**Table 3:** Correlation between QAFRT, Beck, and Zung scales scores in 2016 and 2022 ** Correlation is significant at the 0.01 level (two-tailed). QAFRT = questionnaire for assessing fear of radiotherapy in oncology patients.

Correlations							
		QAFRT score (2016)	QAFRT score (2022)	Beck score (2016)	Beck score (2022)	Zung score (2016)	Zung score (2022)
QAFRT score (2016)	Pearson correlation	1	0.018	0.664^**^	0.037	0.590^**^	0.025
	Sig. (2-tailed)		0.827	0.000	0.650	0.000	0.761
	N	154	154	152	153	154	154
QAFRT score (2022)	Pearson correlation	0.018	1	0.054	0.741^**^	0.020	0.777^**^
	Sig. (2-tailed)	0.827		0.511	0.000	0.808	0.000
	N	154	159	152	158	154	159
Beck score (2016)	Pearson correlation	0.664^**^	0.054	1	0.107	0.722^**^	0.104
	Sig. (2-tailed)	0.000	0.511		.191	0.000	0.201
	N	152	152	152	151	152	152
Beck score (2022)	Pearson correlation	0.037	0.741^**^	0.107	1	0.071	0.828^**^
	Sig. (2-tailed)	0.650	0.000	0.191		0.381	0.000
	N	153	158	151	158	153	158
Zung score (2016)	Pearson correlation	0.590^**^	0.020	0.722^**^	0.071	1	0.075
	Sig. (2-tailed)	0.000	0.808	0.000	0.381		0.353
	N	154	154	152	153	154	154
Zung score (2022)	Pearson correlation	0.025	0.777^**^	0.104	0.828^**^	0.075	1
	Sig. (2-tailed)	0.761	0.000	0.201	0.000	0.353	
	N	154	159	152	158	154	159

## Discussion

The unique research design integrated two prospective studies in two time frames, six years apart, that analyzed fear of radiotherapy and anxiety in patients undergoing radiotherapy treatment at the same center. Thanks to this design, it was possible to investigate how the current health situation has affected the occurrence of the fear and anxiety of oncology patients.

The results of numerous studies show that the COVID-19 pandemic caused an increase in fear and anxiety in oncology patients, but it also influenced decision-making about the regimen of specific oncology treatment [11-14]. In addition, patients had become aware that the COVID-19 pandemic brought about changes in the standard treatment regimens. Current worldwide recommendations for treatment in radiotherapy centers during the COVID-19 pandemic suggested hypofractionated treatment regimens in situations where treatment results are equivalent to standard ones, to reduce the number of hospital visits [15-17]. This fact can enhance fear and uncertainty due to the inability of patients to anticipate the consequences of such changes. Also, there is the ongoing concern that infection of oncology patients with the SARS-CoV-2 virus could lead to a temporary or even permanent suspension in treatment, with long-term consequences for disease control. This is supported by the results of research by Xie and colleagues, who claim that out of 209 patients treated with radiotherapy during the pandemic, 46.4% completed the treatment [18]. Our results indicate that 92 (57.9%) patients experienced SARS-CoV-2 infection before starting radiotherapy treatment, while 91 (57.2%) were vaccinated.

A significant increase in the level of fear and anxiety in patients undergoing radiotherapy during the COVID-19 pandemic compared to 2016 was confirmed and assessed on the basis of the QAFRT questionnaire and Zung's and Beck's scales. The radiotherapy environment, different treatment regimes, concern about potential complications, and the outcome of treatment during emergency medical situations greatly affect the patient's quality of life [19,20]. The application of new, significantly more complex therapeutic modalities increases the retention of patients in radiotherapy centers. Although newer radiotherapy techniques reduce the intensity of radiation toxicity with a remarkable increase in accuracy, they are significantly more demanding in terms of the time required for patient positioning, application of immobilization equipment, creation of a radiotherapy plan, the long-term planning process, and comparative analysis of several radiotherapy plans, while patients spend longer time in radiotherapy centers in the course of their treatment due to the regular implementation of quality assurance procedures [20]. Our study showed the exceptional progress made at the radiotherapy center, which includes the absolute ascendency of new techniques and an increasing amount of information for patients on the treatment regimen, preparation during therapy, and knowledge about potential acute and chronic complications. During the pandemic, 140 (88.1%) patients reported that they had received an adequate explanation about radiotherapy treatment at the first examination by a radiation oncologist, in contrast to the patients treated in 2016, where only 20 (13.0%) of whom responded affirmatively to this question. However, evident progress and the implementation of new treatment techniques did not bring about a reduction in the level of fear they experienced.

Combined EBRT and brachytherapy treatment was applied to patients with gynecological malignancies. The results of earlier studies indicate that the application of EBRT and brachytherapy increases anxiety in female patients [20]. A study conducted by Rades et al. indicates that 70 (57%) female patients experienced fear before brachytherapy, while 50 (41%) showed concern [20]. Here, it is necessary to single out patients who have been prescribed a particular radiotherapy treatment, in which brachytherapy is performed under short-term analgosedation, which greatly increases their fear. The HAPPY study showed that 23 (76.6%) patients actually feel fear of pain during brachytherapy [21]. The results of our research show that the response scores to the QAFRT questionnaire are the highest in both studies in patients treated with EBRT and brachytherapy, but also that the existence of an emergency situation has a strong effect on increasing the fear of patients.

The increase in fear and anxiety in a patient treated with radiotherapy is based on the multifactorial influence of various components that combine the general condition of the patient, the change in lifestyle due to oncological treatment, and factors related to the application of radiotherapy. A study conducted in 2022 showed higher values on the Charlson comorbidity score, higher initial representation of later stages of the disease, and a higher proportion of patients who live alone, which probably accentuated their feelings of loneliness during the period of isolation.

The main limiting factor is the complexity of interpreting the impact of the increase in the level of fear and anxiety in patients treated with radiotherapy in emergency situations. A multi-institutional study with a similar design, as well as a study conducted in the same center with a larger number of subjects after the COVID-19 pandemic would help overcome the current limitations of the study.

## Conclusions

The conducted research showed exceptional differences in the intensity of fear and anxiety in patients treated with radiotherapy in different health situations, as was the case during the COVID-19 pandemic, with a significant impact on the stability of the health system and the challenges to providing standard services. Any emergency situation can increase the fear that the treatment will not be adequate. The highest level of fear was evident in elderly patients who were treated with EBRT and brachytherapy, as well as patients with higher values of the Charlson comorbidity score. Improvement of personnel, technical, and spatial equipment is not always enough to overcome fear and anxiety in oncology patients who are treated with radiotherapy. The findings reinforce the conclusion that patients experiencing a high level of fear and anxiety need organized professional monitoring by psychologists and psychiatrists trained to help oncology patients.

## Appendices

**Table 4:** Questionnaire for assessing fear of radiotherapy in oncology patients

Number	Questions
1.	Do you have a fear that radiation can affect the appearance of a new tumor?
2.	Are you afraid that radiation therapy can damage other organs, which are not subjected to radiotherapy?
3.	Do you have a fear that you will endanger your family because you are in radiotherapy?
4.	Are you afraid that radiotherapy will cause burns at the site of application of radiation?
5.	Are you afraid that radiation therapy will hinder your everyday activities?
6.	Do you have a fear that friends will change their relationship with you because you are being treated with radiotherapy?
7.	Were you afraid when you were told that you would continue the treatment of radiotherapy?
8.	Do you feel disturbed while expecting the application of radiotherapy?
9.	Are you afraid that radiation therapy can cause permanent damage to the region of irradiation?
10.	Do you have a fear that your partner will change their relationship with you because you are being treated with radiotherapy?
11.	Do you think more often than usual about your illness while on radiotherapy?
12.	Do you have a fear that radiotherapy will not be effective against your illness?
13.	Do you have a fear that you have not received all the necessary information about the potential adverse effects of radiotherapy?
14.	Are you afraid to handle electrical appliances, when you are in radiotherapy?
15.	Are you preoccupied with thinking about radiotherapy during the whole day?
